# Ruxolitinib treatment in myelofibrosis and polycythemia vera causes suboptimal humoral immune response following standard and booster vaccination with BNT162b2 mRNA COVID-19 vaccine

**DOI:** 10.3389/fonc.2023.1117815

**Published:** 2023-02-14

**Authors:** Giuseppe A. Palumbo, Daniela Cambria, Enrico La Spina, Andrea Duminuco, Antonio Laneri, Anna Longo, Calogero Vetro, Sebastiano Giallongo, Alessandra Romano, Francesco Di Raimondo, Daniele Tibullo, Cesarina Giallongo

**Affiliations:** ^1^ Dipartimento di Scienze Mediche Chirurgiche e Tecnologie Avanzate “G.F. Ingrassia”, University of Catania, Catania, Italy; ^2^ Unità Operativa Complessa di Ematologia con Trapianto di Midollo Osseo, Azienda Ospedaliero-Universitaria Policlinico “G.Rodolico-San Marco”, Catania, Italy; ^3^ Postgraduate School of Hematology, University of Catania, Catania, Italy; ^4^ Servizio Immuno-Trasfusionale, Azienda Ospedaliero-Universitaria Policlinico “G.Rodolico-San Marco”, Catania, Italy; ^5^ Dipartimento di Chirurgia Generale e Specialità Medico-Chirurgiche, University of Catania, Catania, Italy; ^6^ Dipartimento di Scienze Biomediche e Biotecnologiche, University of Catania, Catania, Italy

**Keywords:** Ruxolitinb, Myelofìbrosis, mRNA vaccine, BNT162.b2, immune response, Polycythemia Vera, COVID-19, SARS-CoV-2

## Abstract

Patients affected by myelofibrosis (MF) or polycythemia vera (PV) and treated with ruxolitinib are at high risk for severe coronavirus disease 2019. Now a vaccine against the virus SARS-CoV-2, which is responsible for this disease, is available. However, sensitivity to vaccines is usually lower in these patients. Moreover, fragile patients were not included in large trials investigating the efficacy of vaccines. Thus, little is known about the efficacy of this approach in this group of patients. In this prospective single-center study, we evaluated 43 patients (30 MF patients and 13 with PV) receiving ruxolitinib as a treatment for their myeloproliferative disease. We measured anti-spike and anti-nucleocapsid IgG against SARS-CoV2 15-30 days after the second and the third BNT162b2 mRNA vaccine booster dose. Patients receiving ruxolitinib showed an impaired antibody response to complete vaccination (2 doses), as 32.5% of patients did not develop any response. After the third booster dose with Comirnaty, results slightly improved, as 80% of these patients produced antibodies above the threshold positivity. However, the quantity of produced antibodies was well below that reached than those reported for healthy individuals. PV patients elicited a better response than patients affected by MF. Thus, different strategies should be considered for this high-risk group of patients.

## 1 Introduction

In February 2020, the World Health Organization (WHO) declared the pandemic for COVID-19 infection caused by the novel coronavirus SARS-CoV-2. The clinical course of the disease is very heterogeneous, spanning from asymptomatic infection to acute respiratory distress syndrome (ARDS) and eventually death (1). Compared to healthy people, patients with comorbidities are considered at higher risk of more aggressive disease and developing severe complications, and myeloproliferative disorders are no exception (2, 3).

On December 2020, results of the BNT162b2 mRNA Covid-19 vaccine clinical trial results were published (4), demonstrating that fully vaccinated people gained a 95% protection against Covid-19, usually reaching a titer >1000 AU/ml (5–7). However, the trial was not conducted on specific fragile patient populations, and data for these subgroups were unavailable. Recently, it has been shown that protection gained by vaccination could be lower in specific immunocompromised patients due to the ongoing treatments and/or the disease itself (8–10). Hemato-oncological patients were among those with blunted vaccination efficacy (5, 11–16).

This is mostly true for lymphoproliferative disorders (5, 11–15, 17), while, in patients with myeloproliferative disorders, a response to vaccination with BNT162b2 like that obtained in healthy individuals has been reported (18–20). In myeloproliferative disorders, a lower Ab response has been reported in MF than in PV or ET (20, 21). Furthermore, ruxolitinib, a JAK 1/2 inhibitor, is widely used in the treatment of MF (22–25) and of hydroxyurea intolerant\resistant PV patients (26, 27). This molecule exerts strong immunosuppressive activity (28) and could be, at least in part, responsible for the inferior efficacy of vaccination. Indeed, in a small number of myeloproliferative patients treated with ruxolitinib, a blunted response to the first (19, 29) and second dose of vaccine (21, 30, 31) was reported. As little data were available in myeloproliferative patients treated with ruxolitinib who had completed the vaccination cycle (2 doses) and a third booster dose, in this study, we investigated whether these patients could reach a protective antibody level against the SARS-CoV-2 virus, as in Italy these patients were granted a fast-track vaccination with BNT162b2 (23).

## 2 Patients and methods

### 2.1 Patients and baseline characteristics

All study participants were administered the two-dose regimen BNT162b2 mRNA vaccine (Corminaty, Pfizer-BioNTech), 30 mcg per dose, by intramuscular injection in the deltoid muscle three weeks apart, as indicated by the Italian national guidelines.

After obtaining informed consent, whole blood sera from the peripheral blood of 43 patients were treated with ruxolitinib. 15 patients were affected by primary MF (PMF), 15 by secondary MF (10 post-PV, PPV-MF, and 5 post-ET, PET-MF) and 13 by PV.

Prognostic risk at first vaccination was calculated with Dynamic International Prognostic Scoring System (DIPSS) (32) for PMF patients: 2 were low, 5 intermediate-1 (Int-1), 7 intermediate-2 (Int-2) and 1 high risk. At the time of booster dose administration, there were no changes in the DIPSS score. Thirteen out of 15 harbored the JAK2 V617F driver mutation, the remaining 2 the CALR mutation.

For secondary MF patients, MYSEC-PM prognostic score (33) was used: 1 patient was low, 5 Int-1, 5 Int-2 and 4 high risk at first vaccination; at booster vaccination, only 2 patients progressed, one from Low to Int-1 and the other from Int-2 to High risk. All PPV-MF harbored the JAK2 V617F mutation together with 3 out of 5 PET-MF, while the remaining 2 had the CALR mutation.

Thirteen patients had a diagnosis of PV, 12 harboring the JAK2 V617F mutation and the remaining one the JAK2 Exon 12. All had received hydroxyurea (HU) treatment before switching to ruxolitinib; for 7 patients this treatment change was due to intolerance, while 6 were resistant to HU according to European Leukemia Network (ELN) consensus criteria (34).

At the time of the first vaccination, the median age was 69 years (range 46-86); for MF patients, the median was 72 years (range 46-86 years), while for PV patients was 64 years (range 50-78 years). The median spleen size was 3 centimeters below the costal margin (range 0-20 cm); in MF patients, the median was 4,6 cm (range 0-20), while in PV patients, it was 0 cm (range 0-2 cm).

After completing the standard vaccination cycle, sera were obtained when they were considered fully vaccinated, at least 14 days (median 36 days, range 14-53) since having received the second dose.

Thirty-nine out of 43 patients received the booster dose, as 2 patients died before, and 2 refused vaccination for personal reasons. The third dose was given at least 32 days and not later than 243 days (median 153 days) after the second dose. Samples were obtained just preceding (the same or the day before) and following the booster administration (median 26 days, range 11-49 days).

Sera were immediately frozen at -20°C until analysis. All demographic data are reported in **Table 1** and **2**.

**Table 1:** Antibody level against SARS-CoV-2 N- and S-proteins in myelofibrosis patients.

UPN	Sex	Age	Diagnosis	Driver mutation	Spleen (cm from costal arch)	DIPSS/MySEC at 1^st^ dose	Median ruxolitinib dose (mg BID)	Days from Ruxolitinib start to 1^st^ vaccination	Days from 2^nd^ dose to testing	Anti-S SARS-CoV2 Ab after 2^nd^ dose	Result after 2^nd^ dose	Days from 2^nd^ to 3^rd^ dose	Anti-S SARS-CoV2 Ab before 3^rd^ dose	DIPSS/MySEC at 3 rd dose	Days from 3^rd^ dose to testing	Anti-S SARS-CoV2 Ab after 3^rd^ dose	Result after 3^rd^ dose
25113	M	72	PMF	JAK2 V617F	1	Int-2	20,0	2025	47	2,0	Negative	237	0.5	Int-2	23	14.2	Positive
19597	F	52	PMF	JAK2 V617F	Splenectomized	Int-1	15,0	3202	33	1409,9	Positive	NA	ND	NA	NA	ND	NA
12930	F	71	PMF	JAK2 V617F	0	Int-2	20,0	107	32	4,8	Negative	150	2,9	Int-2	24	58,9	Positive
42673	M	76	PMF	JAK2 V617F	6	Int-2	15,0	18	44	9,6	Positive	130	1,6	Int-2	28	2,6	Negative
23725	F	46	PMF	JAK2 V617F	6	Low	15,0	189	28	472,1	Positive	166	17,6	Low	21	275,6	Positive
19254	F	67	PMF	JAK2 V617F	0	Int-1	15,0	2682	44	253,6	Positive	152	23,9	Int-1	28	654,7	Positive
987	M	74	PMF	JAK2 V617F	10	Int-2	10,0	26	24	10,0	Positive	133	1,6	Int-2	22	9,6	Positive
16041	F	77	PMF	JAK2 V617F	0	Int-2	10,0	546	45	132,2	Positive	160	0,6	Int-2	30	18,4	Positive
22351	M	77	PMF	CALR	0	Int-2	20,0	1557	43	1333,9*	Positive	169	1,9	Int-2	11	48.3	Positive
33923	M	68	PMF	JAK2 V617F	9	Int-1	25,0	797	21	0,0	Negative	NA	ND	NA	NA	ND	NA
38497	F	70	PMF	JAK2 V617F	6	Int-1	20,0	443	26	6901,5*	Positive	NA	ND	NA	NA	ND	NA
29935	M	75	PMF	CALR	0	Int-2	5,0	426	40	180,3	Positive	178	14,4	Int-2	24	788,4	Positive
12617	M	55	PMF	JAK2 V617F	3	Low	15,0	3288	63	0,3	Negative	166	0,2	Low	28	82,3	Positive
9392	F	61	PMF	JAK2 V617F	6	Int-1	20,0	2992	24	14,0	Positive	167	4,7	Int-1	26	5,3	Negative
20483	M	79	PMF	JAK2 V617F	4	High	12.5	2242	44	3,1	Negative	129	6,7	High	27	12,1	Positive
17044	M	69	PPV-MF	JAK2 V617F	17	Int-1	15,0	793	28	42,7	Positive	169	28,5	Int-1	26	26,8	Positive
5135	M	85	PPV-MF	JAK2 V617F	0	Int-2	15,0	631	29	23,3	Positive	140	12,1	High	29	64,6	Positive
10206	M	66	PPV-MF	JAK2 V617F	9	Int-2	15,0	467	34	2,2	Negative	125	2,4	Int-2	27	3,5	Negative
19206	M	74	PPV-MF	JAK2 V617F	20	Int-1	2,5	2950	46	56,2	Positive	151	6,4	Int-1	27	73,6	Positive
1068	F	76	PPV-MF	JAK2 V617F	0	Int-2	20,0	2687	57	5,5	Negative	143	3,2	Int-2	19	111,5	Positive
42163	M	86	PPV-MF	JAK2 V617F	3	High	15,0	299	19	0,3	Negative	243	0,8	High	21	710,2	Positive
17192	M	76	PPV-MF	JAK2 V617F	10	High	10,0	2127	31	0,4	Negative	148	0,4	High	36	0,4	Negative
31256	F	80	PPV-MF	JAK2 V617F	3	High	10,0	1765	31	33,8	Positive	148	1,7	High	21	83,8	Positive
11185	M	84	PPV-MF	JAK2 V617F	0	High	20,0	2636	16	6,0	Negative	196	2,1	High	21	1,2	Negative
2978	M	69	PPV-MF	JAK2 V617F	0	Int-1	20,0	2703	14	9,7	Positive	167	12,3	Int-1	31	28,9	Positive
0	M	67	PTE-MF	CALR	12	Low	20,0	10	24	80,5	Positive	159	7,7	Int-1	27	30,4	Positive
989	F	66	PTE-MF	JAK2 V617F	5	Int-1	15,0	1127	56	0,0	Negative	56	0,0	Int-1	36	0,0	Negative
3974	M	76	PTE-MF	CALR	1	Int-1	20,0	1970	50	0,3	Negative	169	0,1	Int-1	19	2,5	Negative
26948	F	84	PTE-MF	JAK2 V617F	0	Int-2	20,0	1037	32	0,0	Negative	156	0,8	Int-2	17	518,6	Positive
32127	F	71	PTE-MF	JAK2 V617F	4	Int-2	25,0	767	49	23,4	Positive	175	6,8	Int-2	18	94,8	Positive

Anti-S SARS-CoV2 Ab are expressed as BAU/ml.

*patients with prior exposure to SARS-CoV2 infection and positive IgG against SARS-CoV-2 nucleocapsid proteins (anti-N).

ND, not done; NA, not applicable.

**Table 2:** Antibody level against SARS-CoV-2 N- and S-proteins in polycythemia vera patients.

UPN	Sex	Age	Diagnosis	Driver mutation	Spleen (cm from costal arch)	Intolerant or refractory to HU	Median ruxolitinib dose (mg BID)	Days from Ruxolitinib start to 1^st^ vaccination	Days from 2^nd^ dose to testing	Anti-S SARS-CoV2 Ab after 2^nd^ dose	Result after 2^nd^ dose	Days from 2^nd^ to 3^rd^ dose	Anti-S SARS-CoV2 Ab before 3^rd^ dose	Days from 3^rd^ dose to testing	Anti-S SARS-CoV2 Ab after 3^rd^ dose	Result after 3^rd^ dose
28627	M	64	PV	JAK2 V617F	0	I	15,0	849	50	55,1	Positive	141	9,8	22	204,8	Positive
28075	M	71	PV	JAK2 V617F	0	I	15,0	822	49	101,0	Positive	162	17,3	27	426,3	Positive
40640	M	50	PV	JAK2 V617F	0	I	10,0	53	33	160,9	Positive	145	9,6	28	520,1	Positive
7748	M	74	PV	JAK2 V617F	0	R	15,0	848	33	22,5	Positive	146	10,8	29	882,6	Positive
17076	M	68	PV	JAK2 V617F	2	I	10,0	881	45	42,5	Positive	171	42,5	21	611,2	Positive
9036	M	76	PV	JAK2 V617F	2	R	5,0	213	41	136,2	Positive	125	29.8	49	185,6	Positive
12308	F	56	PV	JAK2 V617F	0	R	15,0	376	14	804,2	Positive	195	50,7	21	1399,5	Positive
26246	M	59	PV	JAK2 V617F	1	R	10,0	3370	55	101,3	Positive	153	25,5	24	1335,3	Positive
12226	M	53	PV	JAK2 V617F	0	I	10,0	645	40	121,4	Positive	152	45,5	21	48,6	Positive
1082	M	52	PV	JAK2 Ex12	0	I	10,0	804	19	3156,6	Positive	NA	ND	NA	ND	NA
19144	F	64	PV	JAK2 V617F	0	I	10,0	445	27	160,4	Positive	110	54,2	21	259,5	Positive
40755	M	73	PV	JAK2 V617F	0	R	10,0	535	32	1,4	Negative	32	1,4	35	2.4	Negative
5307	M	78	PV	JAK2 V617F	0	R	15,0	780	54	11,7	Positive	156	9.0	43	13.1	Positive

Anti-S SARS-CoV2 Ab are expressed as BAU/ml.

ND, not done; NA, not applicable.

### 2.2 Ruxolitinib exposure 

Ruxolitinib administration was started 10-3370 days (median 1236 days) before the first vaccine dose; for MF patients, 10-3288 days before (median 1414 days) while for PV patients 53-3370 days before (median 817 days).

The median ruxolitinib dose at the beginning of the vaccine cycle was 14.7 mg BID (min, max: 2.5, 25 mg); for MF patients, 16,1 mg BID (range 2.5-25 mg), for PV 11,5 mg BID (range 5-15 mg).

There were no significant differences between primary and secondary MF patients, both for the length of exposure and dosages of ruxolitinib.

### 2.3 Antibody level measurements

The SARS-CoV2 virus produces 4 structural proteins, namely envelope, membrane, nucleocapsid and spike, the latter the more immunogenic. Thus, the BNT162b2 mRNA vaccine was designed to induce a strong anti-spike response, resulting in the generation of neutralizing antibodies in >95% of subjects who received 2 doses (35).

The vaccine immunogenicity was evaluated by measuring the serum IgG neutralizing Ab levels against the RBD portion of the spike protein (anti-S), using the IgG II Quant kit (Abbott, Chicago, IL, USA), a chemiluminescent microparticle immunoassay (CIMSA) according to the manufacturer’s instructions on an Architect i2000SR/i4000SR platform. This assay has an optimized sensitivity of 88-98% and a specificity of 100% (36, 37). A value above 7 binding antibody units (BAU), the standardized value according to World Health Organization, was considered as positive.

Vaccine efficacy, in terms of protective immunity, is correlated to the presence of neutralizing antibodies (38–40). In this study, the level of IgG against spike receptor binding domain was used as surrogate markers of neutralizing antibodies because their levels are linearly correlated (41, 42) and as it was deeply shown in animal models (43, 44).

IgG against SARS-CoV-2 nucleocapsid proteins (anti-N) were measured to rule out a prior or ongoing SARS-CoV2 virus infection before vaccination (7). In fact, vaccinated subjects should be anti-S positive and anti-N negative, while patients exposed to the natural virus are anti-S and anti-N positive at the same time (45).

## 3 Results

### 3.1 Antibody level against SARS-CoV-2 N- and S-proteins

Two patients, both affected by PMF, resulted in IgG against SARS-CoV-2 nucleocapsid proteins (anti-N), proving a prior exposure to the virus at the time of the first vaccination, while the remaining 41 were negative. Interestingly, these two subjects showed a high antibody response after vaccination (1333.9 and 6901.5 BAU/ml).

At the completion of the two-dose standard vaccination cycle, the average level of serum IgG neutralizing antibody levels against the RBD portion of the spike protein (anti-S) was 369.5 BAU/ml (range 0-6901 BAUI/ml).

Fourteen out of 43 patients had anti-S antibodies below the threshold positivity; 13/30 MF patients were negative, while only 1/13 PV patients did not mount an antibody response.; 27/43 patients showed less than 60 BAU/ml and 28/44 below 100 BAU/ml.

**Figure 1:**
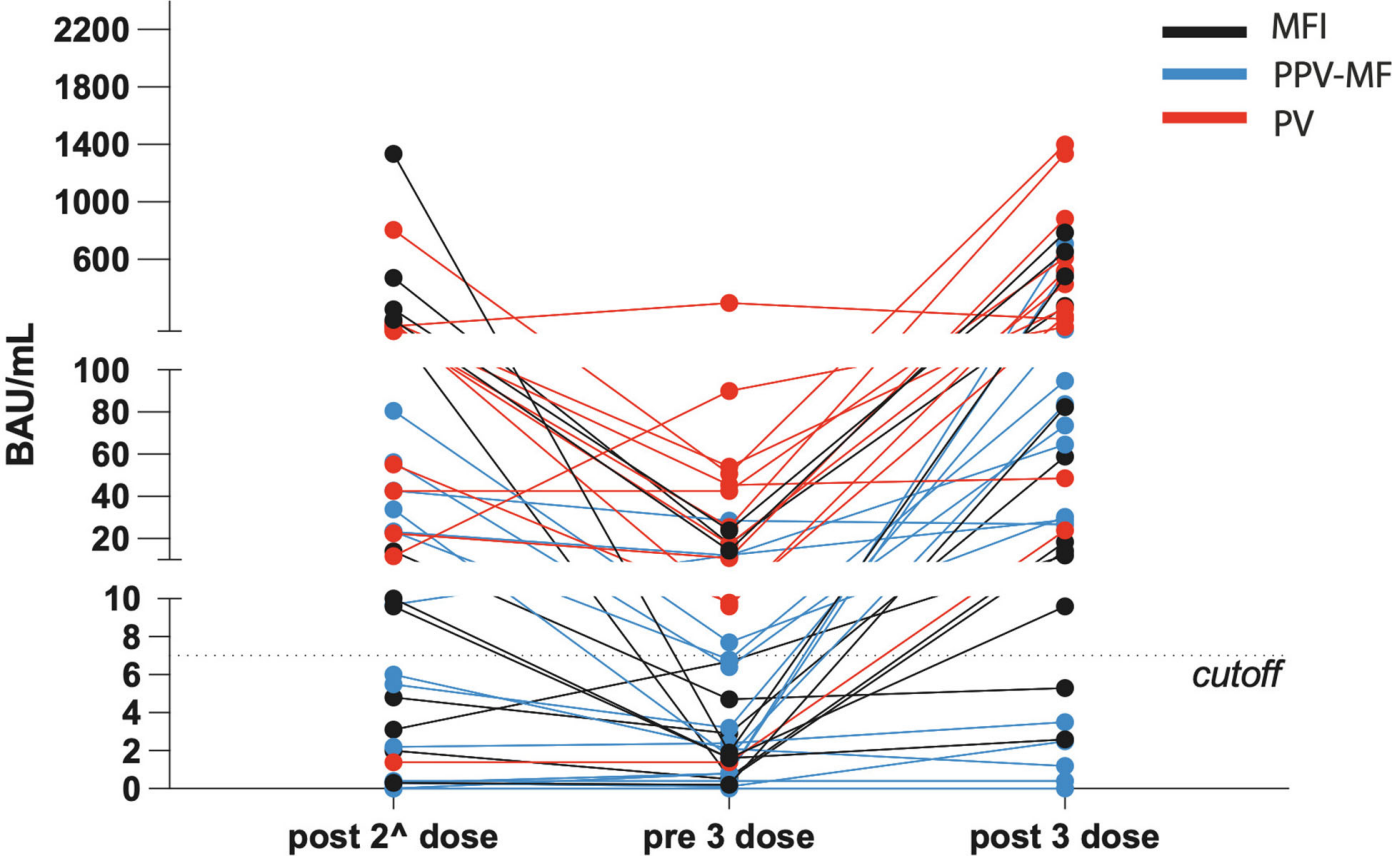
Levels of antibodies against SARS-CoV-2, expressed in BAU/mL, in the context of polycythemia vera, primary and secondary myelofibrosis, during the different time points.

Antibody (Ab) levels before the third booster dose were low (median 12.3, range 0-54.2 BAU/ml). After it, the median value of 272.3 BAU/ml (range 0-1399 BAU/ml was reached. In 8/40 patients (7/27 MF and 1/12 PV), Ab did not raise above the positivity threshold; in 19/39, Ab were below 60 BAU/ml, in 24/39, below 100 BAU/ml. Antibodies development after vaccine doses are reported in **Tables 1**, **2**, and in **Figure 1**.

## 4 Discussion

Vaccination against SARS-CoV2 is considered the most important preventive strategy to counteract severe COVID-19, but its efficacy in hematological malignancy patients seems to be less effective while, on the contrary, these subjects have a high incidence of morbidity and mortality from SARS-CoV-2 infection (2, 17, 46). The blunted response to vaccination has been mainly reported for lymphoproliferative disorders (5, 7, 11, 13–15), while similar responses to those reported in healthy subjects were seen in patients with myeloproliferative disorders (18–20). However, in these patients, a lower response to vaccination could be determined by the type of disease, as it has been reported that MF patients produce fewer antibodies than other bcr\abl negative myeloproliferative disorders (20). The ongoing treatment might also be critical. In particular, ruxolitinib, a widely used JAKi (22–27), is known to downregulate immune response with effects on B, T, dendritic, and NK cells (28) and could be the primary cause of the inferior efficacy of vaccination. Confirming this hypothesis, in patients undergoing ruxolitinib treatment, recently, an impaired response to the first (29) and second dose of vaccine (21, 47) has been demonstrated. Antibodies elicited by vaccination are of key importance to protect subjects from the disease or, in other words, might be able to neutralize the virus (38).

Our study confirmed that ruxolitinib-treated MPN patients who have received 2 standard doses of BNT162b2 show a markedly impaired Ab production. A third booster dose was reported to be able to improve response to vaccination in MF patients (47). This has been shown to be true in our series too. However, although the third booster dose was able to reduce the number of patients who remained fully negative (20% vs. 32.6%), the median Ab value reached was not significantly better, and levels were far from those obtained with the same vaccine dose and schedule in normal subjects. Interestingly, in our and other series (20), MF patients showed a worse response than PV patients, and the explanation could reside in the greater severity of the disease itself (21). Then, it should be taken into consideration that in our series, MF patients showed older age at the time of vaccination, bigger spleen size, longer exposure to ruxolitinib, and at higher median doses, compared to PV patients; each of these factors could play a role in the reduced response to vaccination. Although our results demonstrate a lower humoral response in patients who were assuming ruxolitinib at the same time of Comirnaty administration, caution is needed in concluding that these subjects are not protected against the virus. First, there are not universally validated and accepted antibody cutoffs that correlate with protection against severe COVID-19 disease. A critical point is, in fact, represented by the difference between the antibody positivity, useful to determine whether a subject has been infected by the virus, and the antibody levels that are able to induce a clinically relevant inhibition, that is considered above 50% inhibition (48).

However, in more than 60% of patients, the maximum levels reached are below 100 BAU/ml either after the second and the third vaccine injection. Thus, these patients will have antibodies below the clinically relevant inhibition titer in a short time, considering the known waning in antibody levels over time (42, 49, 50), as it is well known for other coronaviruses. In fact, the decline in total antibodies able to bind the spike protein reflects the decline in neutralizing antibody (41, 42). On the other hand, the disappearance of antibodies reflects the decline in short-lived plasmablasts, while the demonstrated presence of long-lived memory plasma cells could support a rapid response in case of a rechallenge (42). It has been calculated that antibody titers should exponentially fall 250 days after vaccination (40), as it was demonstrated *in vivo* for antibodies after natural infection (51).

In the healthy population, the Ab decline can be counteracted by a strategy based on the administration of a third booster dose. Disappointingly, in our cohort of patients, less than 40% of subjects have anti-spike antibodies above the 100 BAU/ml threshold even after the booster injection and, in any case, well below the levels reached in healthy subjects. Nonetheless, low levels of neutralizing antibodies could persist over time and represent the first line of defense against viral infection (42). Furthermore, surrogate markers of sterilizing immunity have always to be interpreted, taking into consideration the actual scenario with new variants of the virus that keep on emerging (7). And that might need higher Ab levels to be fought.

In addition, it is not known the extent to which humoral response contributes to vaccine efficacy (8). The role of other arms of the immune response, namely cellular immunity elicited by CD4+ and CD8+ T cells, known to be raised by vaccination, must be taken into consideration, and this point is not addressed by our study. In fact, the first subset of T cells is considered a pivot in integrating immune responses, while the latter plays a role in killing cells infected by viruses (52). Both memory and cytotoxic T cells against viruses were shown to last more than 15 years, thus giving the immunized subjects sustained protection over time (53). Indeed, the BNT162b2 vaccine can elicit both humoral and cellular immune responses (54). However, a specific and robust T cell response is more likely to be seen in those patients that elicited a broad functional humoral immune response (50, 55). Thus, antibody levels might be used as a surrogate marker of a good immune response, not limited to B-cells, and can be predictive of protection given by vaccination as the true defensive strength is difficult to assess (38, 39).

It must be considered that the number of participants in our study is relatively low, and no randomized control group has been included. Besides, the limited size of the series does not allow to explore the factors associated with the complete lack of response to the booster dose in around 20% of the patients. Thus, the final word might be given by real-world pharmacovigilance data on the vulnerable population (8). Myeloproliferative patients treated with ruxolitinib should be encouraged to undergo specific vaccination protocols, including prioritization of these patients for a third booster dose that might raise antibody titers (56–59). Furthermore, treatment initiation should be delayed until at least two doses of vaccine have been administered, when clinically possible. For those subjects in which the therapy could not be delayed, newer JAKi, with less immunosuppressive activity, could be considered (28), while for those who are already under ruxolitinib treatment and mount a blunted response, a treatment with Cilgavimab plus Tixagevimab Monoclonal Antibody Cocktail for COVID-19 Prophylaxis could be proposed (23, 60, 61). Anyhow, doctors should be informed that this high-risk group may not be fully protected by vaccination and that risk mitigation, such as social distancing and hygiene measures, should be always implemented by the patients and their caregivers.

## Data availability statement

The original contributions presented in the study are included in the article/supplementary material. Further inquiries can be directed to the corresponding author/s.

## Ethics statement

The study was conducted in accordance with the Declaration of Helsinki and approved by Ethical Committee CT1/AOU Policlinico "G. Rodolico"/San Marco, Catania, Italy. Informed consent was obtained from all subjects involved in the study.

## Author contributions

GAP, DT, and CG designed the research, analyzed the data, and wrote the manuscript. DC, EL, ALo, ALa and AD collected and analyzed the data. CV, AR, AD, and FD analyzed the data and revised the manuscript. All authors contributed to the article and approved the submitted version.
